# Nanozyme-Based Strategies against Bone Infection

**DOI:** 10.34133/research.0605

**Published:** 2025-02-11

**Authors:** Zhenyu Li, Guoqiang Jia, Zheng Su, Chen Zhu

**Affiliations:** Department of Orthopedics, The First Affiliated Hospital of USTC, Division of Life Sciences and Medicine, University of Science and Technology of China, Hefei, Anhui 230001, China.

## Abstract

Nanozymes are a class of nanomaterials that exhibit catalytic functions analogous to those of natural enzymes. They demonstrate considerable promise in the biomedical field, particularly in the treatment of bone infections, due to their distinctive physicochemical properties and adjustable catalytic activities. Bone infections (e.g., periprosthetic infections and osteomyelitis) are infections that are challenging to treat clinically. Traditional treatments often encounter issues related to drug resistance and suboptimal anti-infection outcomes. The advent of nanozymes has brought with it a new avenue of hope for the treatment of bone infections.

## Introduction

Bone infections, or osteomyelitis, remain a marked challenge to treat due to the protective biofilms formed by pathogens and their resistance to conventional antibiotics [1,2]. Bone infection fundamentally disrupts tissue homeostasis through pathological elevation of reactive oxygen species (ROS). During infection, uncontrolled ROS accumulation drives hydrogen peroxide (H₂O₂) to toxic levels of 100 to 1,000 μM while generating excessive superoxide anions (O₂•−) and hydroxyl radicals (•OH) [3]. Such unregulated oxidative stress severely compromises bone healing through dual mechanisms: It directly damages tissue integrity through lipid peroxidation and protein modifications while disrupting the bone remodeling balance by simultaneously suppressing osteoblasts and activating osteoclasts [4]. The key challenge lies in the random, widespread distribution of these harmful ROS species throughout the infected tissue, which creates an environment that impairs both immune function and tissue regeneration. This pathological oxidative landscape underscores the critical need for precise, localized ROS modulation strategies that can both eliminate pathogens and support healing processes.

The advent of nanozymes offers novel approaches, whereby their catalytic activity is exploited to generate antimicrobial agents directly within infected sites [5,6]. Nanozymes, which are typically metal-based nanomaterials, have been observed to possess enzyme-mimicking properties, including the ability to perform the functions of peroxidase, catalase (CAT), and superoxide dismutase (SOD) [7–9]. These properties facilitate not only direct combat against pathogens but also modulation of the local microenvironment, thereby aiding in soft tissue recovery and bone regeneration, as illustrated in Figure [10–13].

**Figure.**
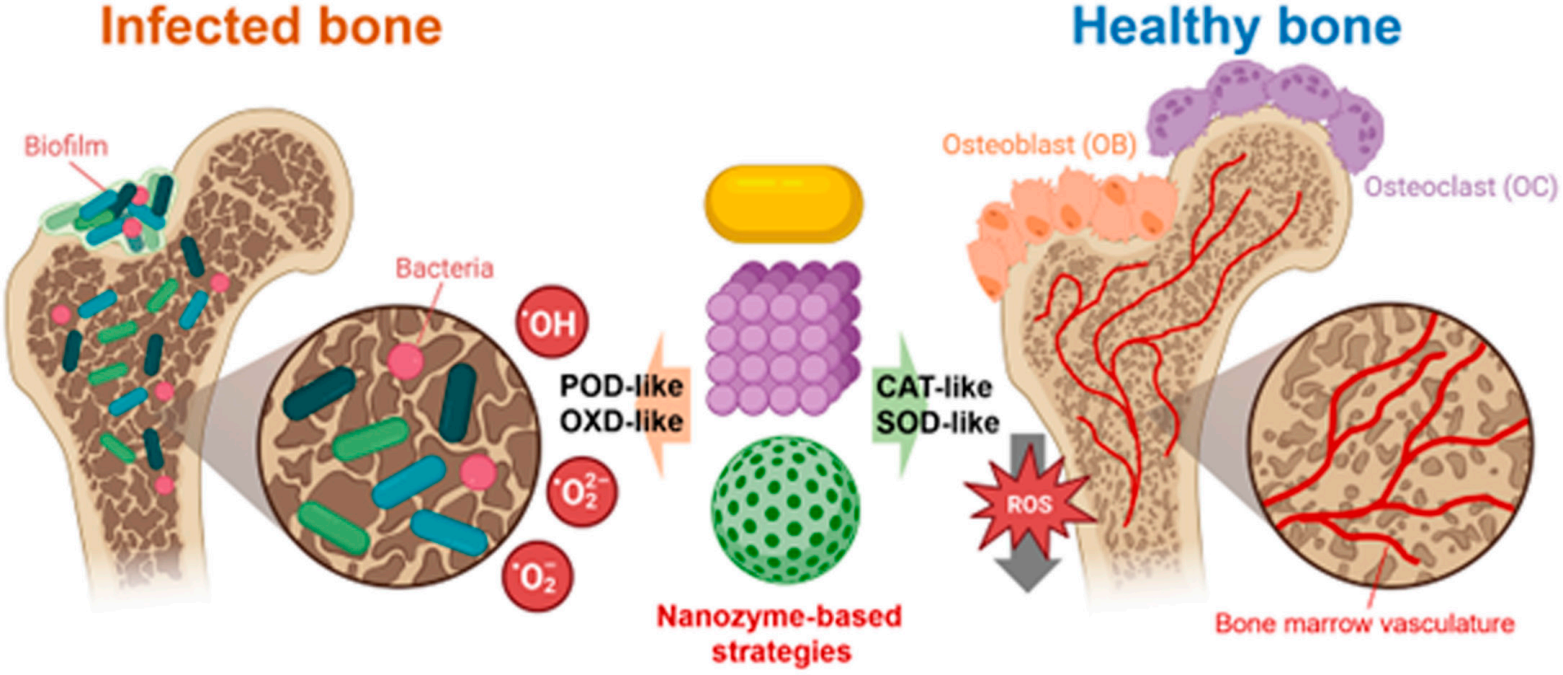
Nanozyme-based strategies for bacterial killing and bone regeneration. (The figure was constructed using graphical assets obtained from Biorender.com.)

The unique structural and physiological characteristics of bone tissue make nanozymes particularly promising for treating bone infections. While soft tissues generally respond well to standard antimicrobial therapies, bone’s dense mineralized matrix and limited vasculature create substantial barriers for traditional drug delivery systems, further complicated by the distinctive acidic microenvironment (pH 5.5 to 6.5) that develops during osteomyelitis [14]. Nanozymes address these challenges through their optimally engineered dimensions (10 to 100 nm) that enable efficient bone penetration, enhanced catalytic activity in acidic conditions, and remarkable dual functionality in both bacterial elimination and osteogenic regeneration promotion, simultaneously targeting both the infectious and reconstructive aspects of bone healing [15].

## Antibacterial Mechanisms of Nanozymes

Nanozymes exert their antibacterial effects primarily by mimicking the activity of natural enzymes and catalyzing specific reactions that generate bactericidal agents such as ROS [16,17] or hypochlorous acid (HClO) [18]. These substances effectively interfere with bacterial cell structures and metabolic functions, leading to bacterial cell death, particularly against multidrug-resistant (MDR) strains [19–23]. The antibacterial efficacy of nanozymes depends on 2 key features of their catalytic reactions, such as enzyme-mimicking activity and ROS production [24]. The catalytic activities are highly condition-dependent: Optimal peroxidase-like activity of Fe₃O₄ nanoparticles occurs at pH 3.5 to 4.0 and 37 °C, with *K*_m_ values of 3.84 mM for H₂O₂ and 0.098 mM for TMB substrate, achieving >99% bacterial killing rates at H₂O₂ concentrations of 0.1 to 1 mM. At physiological pH 7.4, the activity decreases by approximately 60%, necessitating higher H₂O₂ concentrations (>5 mM) for effective antibacterial action [25]. Certain nanozymes possess enzyme-like activities similar to those of natural peroxidases, such as horseradish peroxidase (HRP), enabling them to catalyze the decomposition of H₂O₂ under physiological conditions to produce ROS [26]. These ROS, including hydroxyl radicals (•OH), singlet oxygen (^1^O₂), and superoxide anions (O₂•−), have potent oxidative capabilities that can degrade bacterial cell membranes, DNA, proteins, and other cellular components, ultimately leading to bacterial death [27]. For example, Fe₃O₄ nanoparticles exhibit peroxidase-like activity, generating •OH in the presence of low concentrations of H₂O₂, effectively killing bacteria [17]. Similarly, manganese oxide (MnO₂) nanoparticles can modulate different oxidation states (Mn^2+^/Mn^4+^) to catalyze ROS production in the presence of H₂O₂ [28]. Under specific conditions, Mn^2+^-based nanozymes show optimal oxidase-like activity at pH 5.0 and 25 °C with a *K*_m_ value of 0.21 mM for TMB, while Mn^4+^-based variants demonstrate enhanced peroxidase-like activity (*K*_m_ = 0.87 mM for H₂O₂) at pH 4.0 and 40 °C. The catalytic efficiency (*k*_cat_/*K*_m_) shows a 3-fold increase as temperature rises from 25 °C to 40 °C, particularly beneficial at infection sites with elevated local temperatures [29]. In addition, nanozymes can mimic the activity of natural HClO-generating enzymes, catalyzing the conversion of chloride ions to HClO [18]. This compound rapidly disrupts bacterial cell walls and induces cell death due to its strong oxidative properties.

## Effect of Nanozymes on Bacterial Biofilms

Nanozymes achieve broad-spectrum antibacterial effects by generating ROS or HClO that target a diverse array of bacterial species [30–32]. These agents show activity against both gram-positive bacteria, such as *Staphylococcus aureus*, and gram-negative bacteria, including *Escherichia coli*. Notably, they also show activity against MDR strains. The cell wall architectures of MDR bacteria often pose important challenges to antibiotic permeation [33–36]. Nanozymes can bypass these antibiotic targets by inducing nonselective oxidative stress mechanisms against bacteria, allowing effective eradication of resistant strains [37,38]. The nonspecific destruction of bacterial cell structures by chemically generated ROS or HClO makes it difficult for bacteria to develop resistance through genetic mutation. In addition, the rapid production of ROS and their potent oxidative effects lead to multiple types of damage within bacterial cells, accelerating bacterial death and further reducing the likelihood of resistance emergence [39–41]. Despite the promising potential of nanozymes in antibacterial applications, several challenges remain. Issues relating to their stability, toxicity, and biocompatibility in vivo require further investigation. In particular, the SOD- and CAT-like activities of nanozymes require careful consideration in bone infection treatment. These antioxidant activities may reduce ROS levels and potentially compromise antibacterial efficacy. Therefore, when designing nanozymes for bone infections, priority should be given to enhancing their peroxidase-like activity to ensure sufficient antibacterial effects, while restricting antioxidant functions to the healing phase.

Bacterial biofilms represent a structural protective barrier composed of polysaccharides, proteins, and nucleic acids secreted by bacteria at infection sites [1,22,23,42,43]. These biofilms are prevalent in chronic bone infections and serve not only to shield bacteria from antibiotic action and host immune responses but also to facilitate the development of bacterial resistance, which substantially complicates treatment efforts [44–47]. Some nanozymes possess sharp edges, such as true spine-like arrays, which enable them to not only chemically disrupt biofilms but also physically weaken their stability [48–51]. The elevated surface area and nanoscale composition of these particles enable them to penetrate biofilms and directly disrupt their physical structure, thereby impairing bacterial adhesion.

## Combined Application of Nanozymes and Traditional Antibiotics

The coapplication of nanozymes with traditional antibiotics represents a marked avenue of research in the treatment of bone infections. The integration of nanozymes with conventional antibiotics can markedly enhance antimicrobial efficacy, thereby increasing treatment effectiveness. This is achieved by combining the antibacterial properties of nanozymes with the therapeutic mechanisms of conventional antibiotics. The use of nanozymes can mitigate the development of bacterial resistance by disrupting the mechanisms employed by bacteria to evade treatment, such as the formation of biofilms and the interference with signaling pathways. This, in turn, enhances the efficacy of antibiotics [52,53]. Certain nanozymes can influence bacterial quorum-sensing mechanisms, reducing bacterial resistance to antibiotics and increasing the effectiveness of traditional therapies [54–56]. The incorporation of nanozymes may facilitate the intracellular accumulation of antibiotics by modulating cell membrane permeability, thereby enabling antibiotics to enter bacterial cells with greater efficiency and enhancing their antibacterial effects. The use of nanozymes in conjunction with traditional antibiotics allows for a reduction in the dosage of the latter, thereby limiting the impact on normal bacterial populations and reducing the selective pressure for resistant strains. The combination of nanozymes and traditional antibiotics provides new avenues for the treatment of bone infections. The incorporation of different types of nanozymes and antibiotics in multitarget treatment strategies allows for a comprehensive approach to addressing complex bacterial infections, thereby improving the success rate of treatments.

## Intelligently Responsive Nanozymes

Intelligently responsive nanozymes represent a class of nanomaterials that are capable of dynamically adjusting their catalytic activity in response to specific stimuli within their microenvironment. These stimuli may include changes in pH, H₂O₂ concentration, temperature, or external stimuli such as light, sound, heat, and electricity [52,57,58]. These nanozymes can achieve targeted activation in the unique pathological conditions present at infection sites, increasing their antibacterial activity while maintaining low activity in healthy tissues, thereby minimizing side effects [10,16,26]. This intelligent responsive design provides greater specificity and safety for the use of nanozymes, particularly in the treatment of complex infections [59]. The core functionality of intelligent responsive nanozymes lies in their structural design, which enables them to respond to specific environmental triggers and alter their catalytic capabilities [60–62]. These responses are often associated with characteristics that differ between pathological environments and healthy tissues, such as acidic conditions and elevated H_2_O_2_ concentrations [58,63]. Su et al. designed a biofilm microenvironment-responsive double-layered metal-organic framework bionanocatalysts composed of MIL-100 and CuBTC. As an activable photothermal agent, 2,2′-azino-bis (3-ethylbenzothiazoline-6-sulfonic acid) (ABTS) was loaded into the mesopores of MIL-100, while glucose oxidase (GOx) was encapsulated within the framework of CuBTC, thus yielding a (MIL-100-ABTS)@(CuBTC-GOx) bionanocatalyst. Once the bionanocatalyst reached the acidic biofilm microenvironment, the outer CuBTC degraded to release GOx for catalyzing the conversion of glucose into H_2_O_2_ and gluconic acid, which increased the acidification of biofilm microenvironment to promote the degradation of CuBTC and accelerate the release of GOx/ABTS. Further, HRP-mimicking MIL-100 activated photothermal effect of MACG by ABTS oxidation in the presence of self-supplied H_2_O_2_. Upon near-infrared laser irradiation, the generated sufficient heat flow could loosen the dense biofilm via extracellular DNA damage and open the pore channels in the biofilm to reduce its resistance to •OH. Then, the Cu ion released from the degraded CuBTC depletes glutathione and catalyzed the splitting of extra H_2_O_2_ into •OH to kill sessile bacteria of inner biofilms without huge resistance [26]. In addition, infections and inflammatory responses often result in localized increases in temperature, which can trigger nanozyme activation. External stimuli such as near-infrared light and ultrasound can induce nanozymes to produce ROS. For example, Bai et al. recently developed a copper single-atom nanozyme system (CuNx-CNS) that demonstrates superior multienzyme activities and NIR-II responsiveness particularly suitable for deep tissue infections. In their work, they designed the system with atomically dispersed copper sites anchored on ultrathin 2D porous N-doped carbon nanosheets, with tunable N coordination numbers (*x* = 2 or 4). This nanozyme system exhibits triple enzyme-like activities (peroxidase, CAT, and oxidase), enabling efficient ROS generation through multiple pathways. Notably, their research showed that increasing the N coordination number from 2 to 4 enhances the multienzyme activities due to optimized electron structure. The system’s strong absorption in the second near-infrared (NIR-II) biowindow enables deeper tissue penetration, facilitating both enhanced ROS generation and photothermal treatment in deep tissues, making it particularly effective against MDR bacteria and stubborn biofilms in both superficial and deep implant-related infections [24]. In addition, different bacterial species have specific microenvironmental characteristics and metabolites that can also be used as sources of triggers for nanozymatic catalytic reactions [26]. Through these intelligent response mechanisms, nanozymes hold great promise for advancing targeted therapeutic strategies, increasing the efficacy of infection treatments while minimizing adverse effects on surrounding healthy tissues.

## Promotion of Bone Regeneration by Nanozymes

The promotion of bone regeneration represents an important potential avenue for the utilization of nanozymes in the management of bone infections [64,65]. In addition to their antibacterial properties, nanozymes have the potential to facilitate the repair and regeneration of bone tissue by regulating oxidative stress within the body and promoting osteoblast proliferation and differentiation [66,67]. Bone infections and injuries are frequently accompanied by heightened inflammatory responses and oxidative stress. Consequently, modulation of this microenvironment is of paramount importance for the promotion of bone regeneration [68]. Bone repair and regeneration involves complex cellular behavior and microenvironmental regulation, including the synergistic actions of osteoblasts (bone formation), osteoclasts (bone resorption), and mesenchymal cells [69]. During the healing process of bone infections or defects, excessive ROS can exacerbate tissue damage while inhibiting the proliferation and differentiation of osteoblasts, thereby hindering bone tissue repair [70,71]. It is therefore of the utmost importance to control ROS levels and mitigate the effects of oxidative stress on the bone repair microenvironment. Specific nanozymes have the capacity to emulate the function of natural SOD, facilitating the conversion of O₂•− into H₂O₂ and oxygen (O₂), which serves to mitigate the damaging effects of oxidative stress and safeguard osteoblasts [72–75]. Nanozymes that exhibit CAT-like activity can further convert excess H₂O₂ into H₂O and O₂, thereby reducing inflammatory responses and enhancing the bone repair microenvironment [76–80]. In addition to regulating oxidative stress, nanozymes with metal ions can directly promote osteoblast differentiation and mineralization by modulating cellular signaling pathways [81–84]. These multifaceted mechanisms have led to the emergence of nanozymes as a promising agent for advancing bone regeneration, which ultimately improves outcomes in the treatment of bone infections and injuries.

The specific advantages of nanozymes in bone infection treatment are particularly evident in their interaction with bone tissue. Their nanoscale dimensions allow them to penetrate the hierarchical structure of bone tissue, including micropores (10 to 20 μm) and canaliculi (0.1 to 1 μm), enabling better distribution throughout infected sites. Furthermore, the acidic microenvironment (pH 5.5 to 6.5) characteristic of osteomyelitis actually enhances the peroxidase-like activity of certain nanozymes, such as Fe_3_O_4_ nanoparticles, making them more effective precisely where needed [85]. The presence of elevated H_2_O_2_ levels (100 to 1,000 μM) in infected bone tissue provides an ideal substrate for nanozyme-mediated ROS generation. Additionally, nanozymes can specifically bind to hydroxyapatite in bone tissue through surface modification with bisphosphonate groups, enabling targeted and prolonged therapeutic effects. This bone-specific targeting, combined with their ability to modulate the RANKL (receptor activator of nuclear factor kappa-Β ligand)/RANK (receptor activator of nuclear factor kappa-Β)/OPG (osteoprotegerin) pathway crucial for bone homeostasis, makes nanozymes particularly suitable for treating bone infections [86].

## Application of Nanozymes in the Diagnosis of Bone Infections

The utilization of nanozymes in the diagnosis of bone infections is a promising avenue of research, particularly given the enzyme-mimicking properties of nanozymes, which facilitate the sensitive and selective detection of bacterial pathogens, biomarkers, and infection-related by-products [87–90]. In the context of bone infections, traditional diagnostic methods such as microbial cultures frequently encounter limitations due to the sluggish growth rates of pathogens and the formation of biofilms that serve to protect bacteria. Nanozymes have the potential to provide solutions by accelerating the detection of pathogens and improving sensitivity [91,92]. To illustrate, nanozymes exhibiting peroxidase-like activity can facilitate reactions with H₂O₂ in the presence of chromogenic substrates, resulting in discernible color changes that indicate the presence of an infection [93]. The integration of nanozymes with biosensors has further enhanced their capacity to provide real-time data on infection status, thereby facilitating the implementation of timely clinical interventions. These developments illustrate the dual diagnostic and therapeutic potential of nanozymes in the management of bone infections. Further research into nanozyme-based detection may facilitate earlier diagnosis, reduce the necessity for invasive sampling, and facilitate the monitoring of treatment efficacy in the management of bone infections.

## Long-Term Safety Concerns

Long-term safety concerns specifically relate to several aspects of nanozyme behavior in vivo. The catalytic nature of nanozymes means they can potentially maintain activity for extended periods, raising questions about chronic ROS exposure even at low levels. Metal-based nanozymes may undergo gradual degradation, leading to accumulation of metal ions that could affect bone mineralization processes or cellular function [94]. The interaction between nanozymes and the bone extracellular matrix over time requires careful evaluation, as changes in nanozyme surface properties during long-term residence might alter their activity or distribution.

## Outlook

The implementation of personalized treatment is becoming an increasingly crucial aspect in the management of bone infections, particularly when integrated with modern precision medicine technologies that facilitate the development of bespoke therapeutic strategies. The identification of specific biomarkers and the utilization of gene editing techniques permit the design of nanozymes that are capable of targeting a range of bone infection types and severities. This approach enhances both the efficacy and safety of treatments. As our comprehension of individual differences grows, precision medicine places an emphasis on the consideration of patients’ genetic backgrounds, pathological features, and environmental factors in therapeutic approaches. In the treatment of bone infections, this personalized approach allows for the optimization of strategies based on the nature of the infection, the type of pathogen involved, and the patient’s overall health status. Personalized treatment is not confined to the utilization of static protocols; it can also employ real-time monitoring technologies to facilitate dynamic adjustments to treatment efficacy. By continuously monitoring changes in the infection, clinicians can promptly modify the dosage and release the timing of nanozymes in accordance with the patient’s response, thereby achieving a tailored therapeutic outcome. Moreover, intelligent release systems that integrate biosensor technologies with smart nanozyme platforms can facilitate real-time monitoring of intracellular ROS levels, pH, or concentrations of specific biomarkers. These systems are capable of automatically adjusting the release and activity of nanozymes based on these metrics, thereby enabling precision treatment in the context of infection management. This innovative approach has the potential to revolutionize the treatment landscape for bone infections, offering the possibility of more effective and personalized care that is tailored to the distinct clinical circumstances of each patient. Future research should aim to elucidate the mechanisms underlying nanozymes and corroborate their clinical utility, thus propelling this emerging field forward.
